# Supplementary material for: JAK inhibitors dampen activation of interferon-stimulated transcription of ACE2 isoforms in human airway epithelial cells
Source: Commun Biol. 2021 Jun 2;4:654. doi: 10.1038/s42003-021-02167-1 (PMC8172581; doi:10.1038/s42003-021-02167-1)
Supplement: Supplementary file 2 — Supplementary Information [file 42003_2021_2167_MOESM2_ESM.pdf]

Supplementary Information

JAK inhibitors dampen activation of interferon-stimulated transcription of ACE2 isoforms in human airway epithelial cells

Hye Kyung Lee[1], Olive Jung[2, 3], and Lothar Hennighausen[1]

[1]Laboratory of Genetics and Physiology, National Institute of Diabetes, Digestive and Kidney Diseases, National Institutes of Health, Bethesda, Maryland 20892.

[2]Division of Preclinical Innovation, National Center for Advancing Translational Sciences, National Institutes of Health, Bethesda, Maryland 20892.

[3]Biomedical Ultrasonics & Biotherapy Laboratory, Institute of Biomedical Engineering, Department of Engineering Science, Old Road Campus Research Building, University of Oxford, Headington, Oxford OX3 7DQ, UK.

*Correspondence to: L.H (lotharh@niddk.nih.gov) and H.K.L (hyekyung.lee@nih.gov)

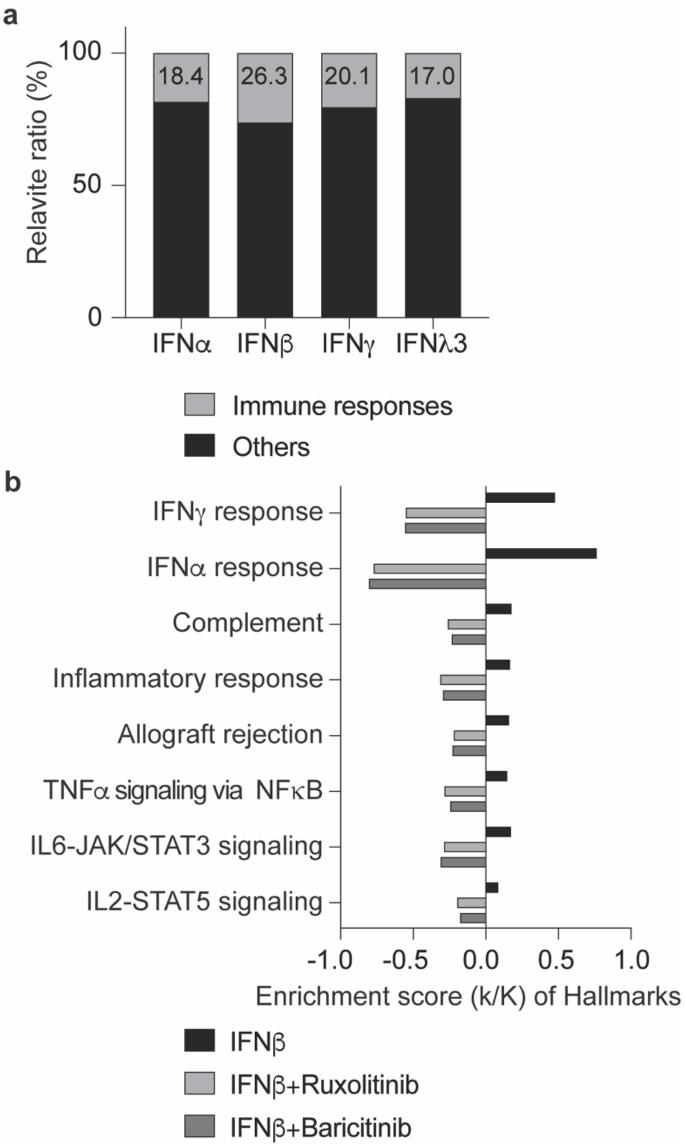

Supplementary Figure 1. Activation of immune response genes by Interferons in human primary airway epithelial cells (SAECs) is mitigated by JAK inhibitors. a. Genes induced significantly by IFNα, β, γ and λ3 were significantly enriched in Hallmark Gene Sets (FDR q-value < 0.005). Immune response genes accounted for 18.4%, 26.3%, 20.1% and 17% of the upregulated genes. **b.** Expression of immune pathway genes induced by IFNβ was mitigated by the JAK inhibitors, ruxolitinib and baricitinib.

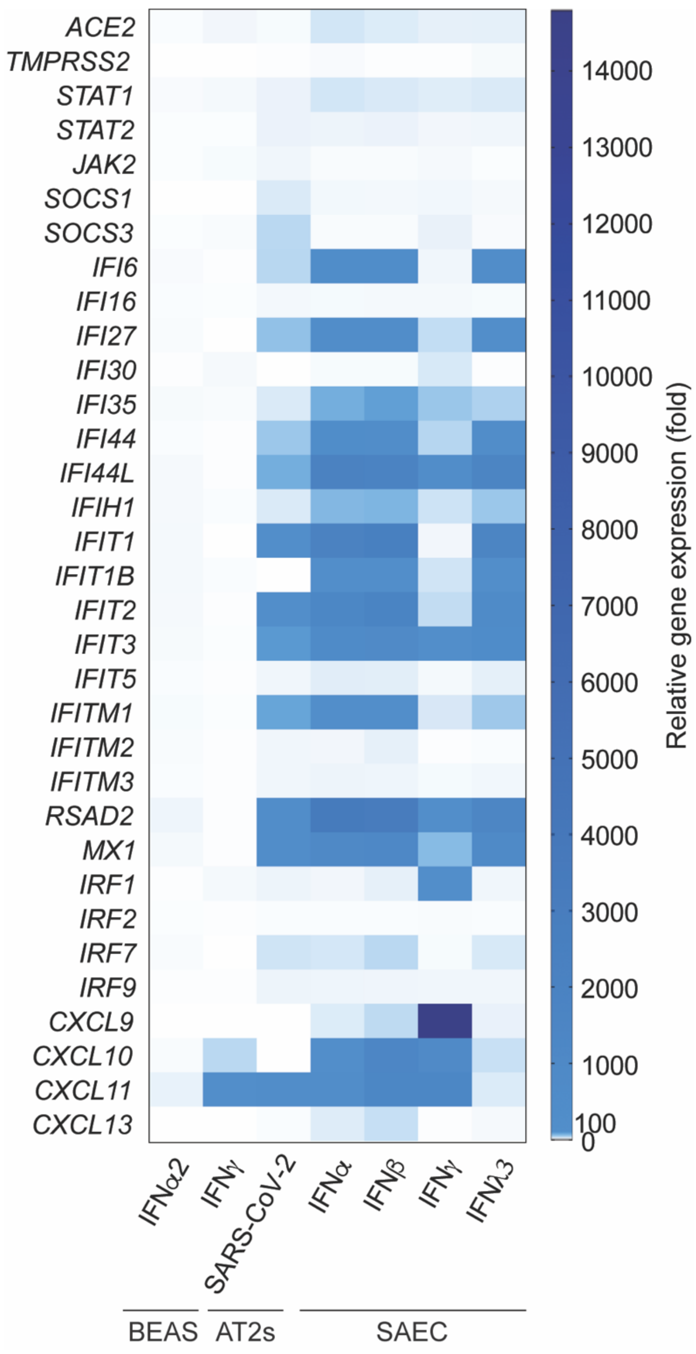

Supplementary Figure 2. Comparison of Interferon-response genes in human bronchial epithelium, primary alveolar epithelial type-2 cells and primary airway epithelium. Expression of genes related to COVID-19 and cytokine signaling from interferons treated BEAS-2B cells (GSE148829)[1], SARS-CoV-2 infected AT2s[2] (GSE152586) and interferons treated SAECs (this study) was displayed by heatmaps.

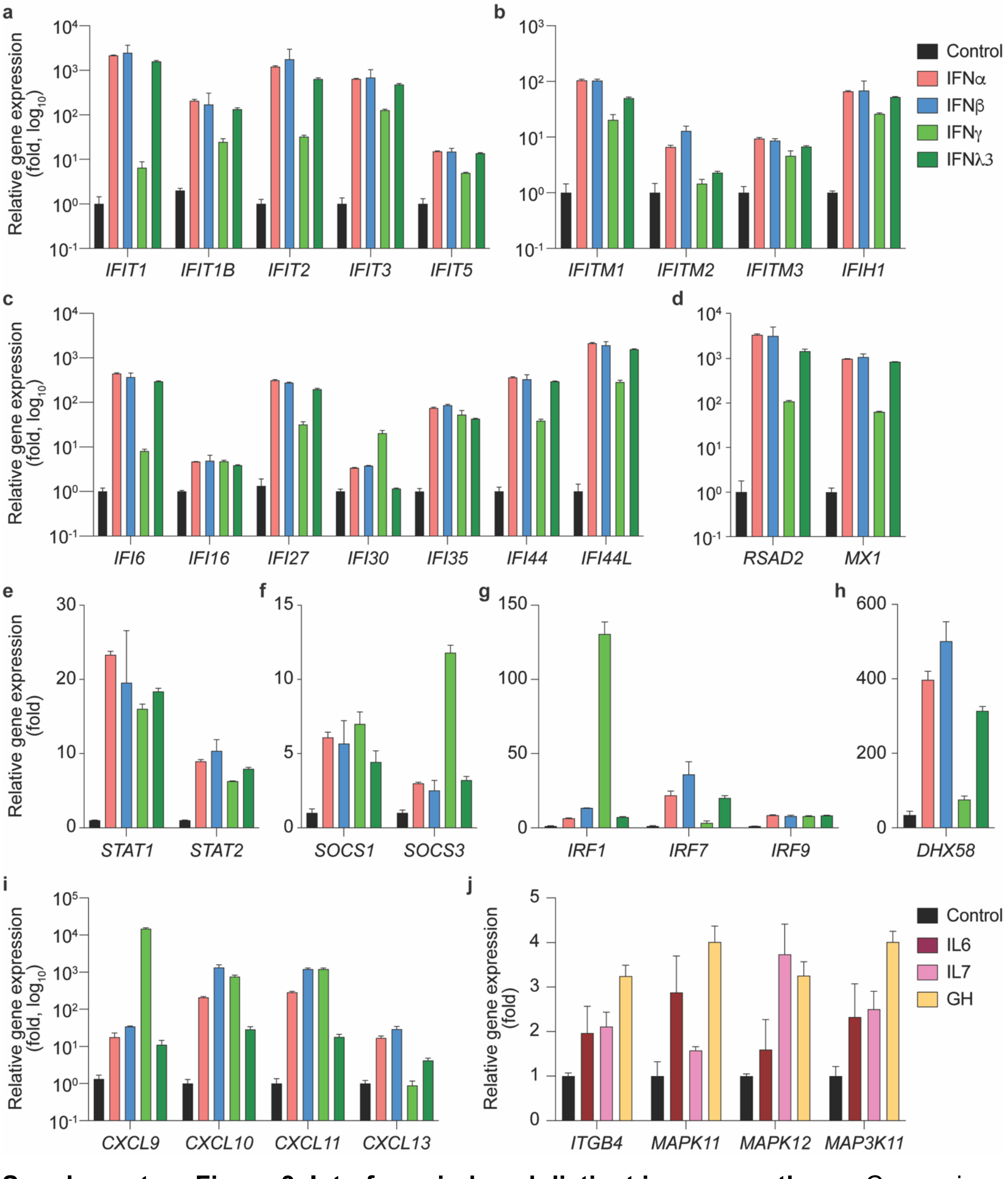

Supplementary Figure 3. Interferon induced distinct immune pathway. Comparison of relative normalized gene expression levels of representative genes between control (n=4) and IFNs-treated (n=3) groups. Results are shown as the means ± s.e.m. of independent biological replicates.

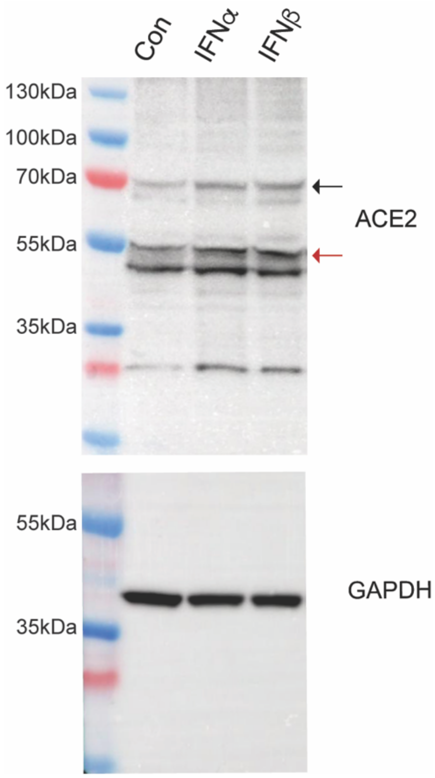

Supplementary Figure 4. Interferon induced distinct immune pathway. Comparison of relative normalized gene expression levels of representative genes between control (n=4) and IFNs-treated (n=3) groups. Results are shown as the means ± s.e.m. of independent biological replicates.

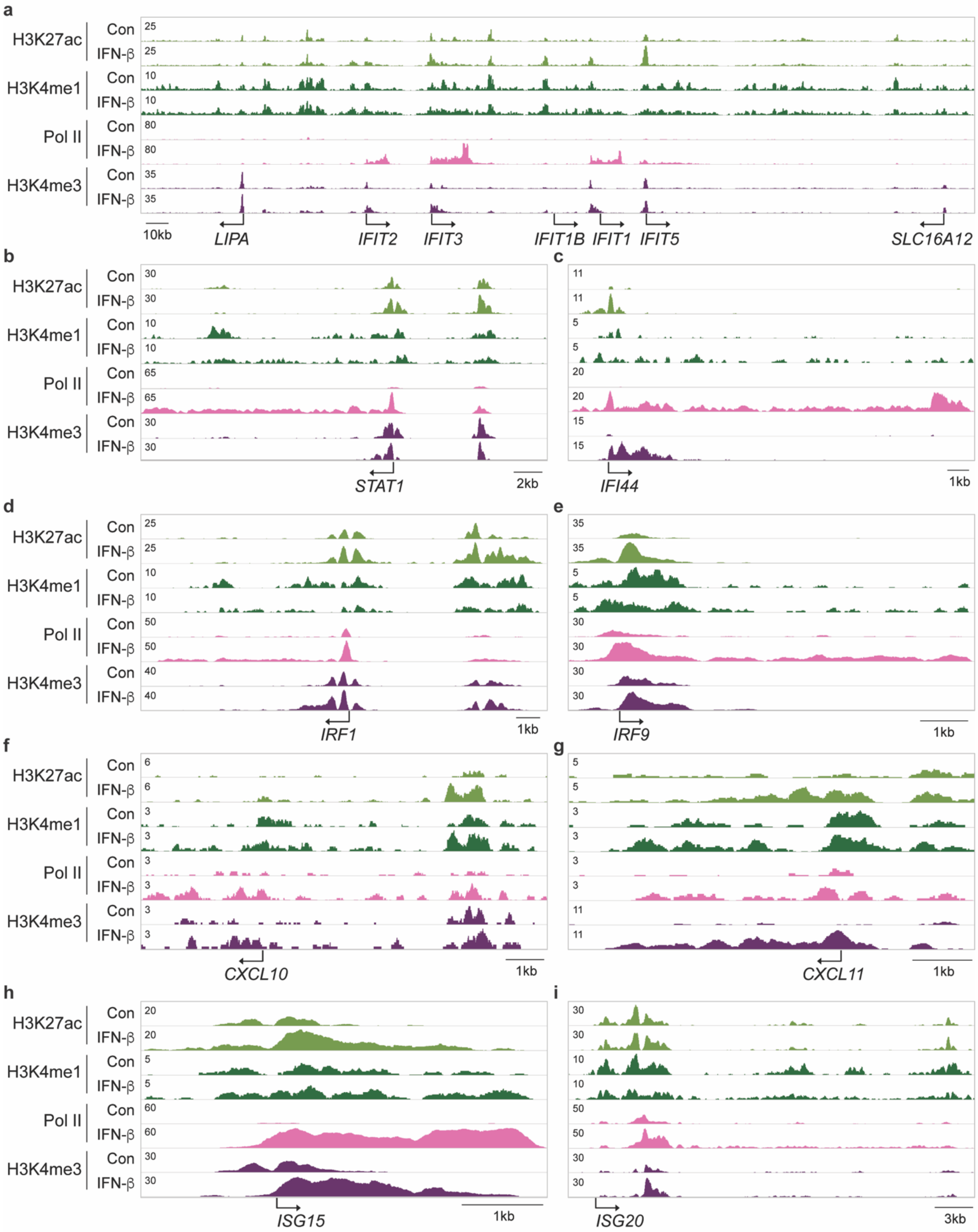

Supplementary Figure 5. IFN induces ACE2 and dACE2 isoform expression. ACE2 and putative dACE2 proteins were detected by western blot in IFNα/β-treated cells.

Supplementary References

1. Ziegler, C.G.K. et al. SARS-CoV-2 Receptor ACE2 Is an Interferon-Stimulated Gene in Human Airway Epithelial Cells and Is Detected in Specific Cell Subsets across Tissues. Cell 181(5):1016-1035.e19 2020).

2. Katsura, H. et al. Human Lung Stem Cell-Based Alveolospheres Provide Insights into SARS-CoV-2-Mediated Interferon Responses and Pneumocyte Dysfunction. Cell Stem Cell 27, 890-904.e8 (2020)
