# Supplementary material for: JAK inhibitors dampen activation of interferon-stimulated transcription of ACE2 isoforms in human airway epithelial cells
Source: Commun Biol. 2021 Jun 2;4:654. doi: 10.1038/s42003-021-02167-1 (PMC8172581; doi:10.1038/s42003-021-02167-1)
Supplement: Supplementary file 3 — Description of Additional Supplementary Files [file 42003_2021_2167_MOESM3_ESM.pdf]

Description of Additional Supplementary Files

File Name: Supplementary Data 1

Description: mRNA levels of genes associated with the pan JAK-STAT pathway in SAECs.

File Name: Supplementary Data 2

Description: List of all genes with normalized read counts in each replicate at Control and IFNa treated SAECs, $\log_2$ fold change), p-value and adjusted p-value as well as upregulated gene list and GSEA analysis.

File Name: Supplementary Data 3

Description: List of all genes with normalized read counts in each replicate at Control and IFNb treated SAECs, $\log_2$ (fold change), p-value and adjusted p-value as well as upregulated gene list and GSEA analysis.

File Name: Supplementary Data 4

Description: List of all genes with normalized read counts in each replicate at Control and IFNg treated SAECs, $\log_2$ (fold change), p-value and adjusted p-value as well as upregulated gene list and GSEA analysis.

File Name: Supplementary Data 5

Description: List of all genes with normalized read counts in each replicate at Control and IFNl3 treated SAECs, $\log_2$ (fold change), p-value and adjusted p-value as well as upregulated gene list and GSEA analysis.

File Name: Supplementary Data 6

Description: List of all genes with normalized read counts in each replicate at Control and IL6 treated SAECs, $\log_2$ (fold change), p-value and adjusted p-value as well as upregulated gene list and GSEA analysis.

File Name: Supplementary Data 7

Description: List of all genes with normalized read counts in each replicate at Control and IL7 treated SAECs, $\log_2$ (fold change), p-value and adjusted p-value as well as upregulated gene list and GSEA analysis.

File Name: Supplementary Data 8

Description: List of all genes with normalized read counts in each replicate at Control and GH treated SAECs, $\log_2$ (fold change), p-value and adjusted p-value as well as upregulated gene list and GSEA analysis.

File Name: Supplementary Data 9

Description: List of genes induced by IFNa, b, g and l. Expression prior to and after interferon stimulation and upon treatment with ruxolitinib and baricitinib is shown.

File Name: Supplementary Data 10

Description: List of all genes with normalized read counts in each replicate at IFNb and baricitinib with IFNb, treated SAECs, log_2 (fold change), p-value and adjusted p-value as well as upregulated gene list and GSEA analysis.

File Name: Supplementary Data 11

Description: List of all genes with normalized read counts in each replicate at IFNb and ruxolitinib with IFNb, treated SAECs, log_2 (fold change), p-value and adjusted p-value as well as upregulated gene list and GSEA analysis.
