# Supplementary material for: JAK inhibitors dampen activation of interferon-stimulated transcription of ACE2 isoforms in human airway epithelial cells
Source: Commun Biol. 2021 Jun 2;4:654. doi: 10.1038/s42003-021-02167-1 (PMC8172581; doi:10.1038/s42003-021-02167-1)
Supplement: Supplementary file 15 — Reporting Summary [file 42003_2021_2167_MOESM15_ESM.pdf]

nature research

Corresponding author(s): Lothar Hennighausen

Last updated by author(s): Mar 14, 2021

Reporting Summary

Nature Research wishes to improve the reproducibility of the work that we publish. This form provides structure for consistency and transparency in reporting. For further information on Nature Research policies, see our Editorial Policies and the Editorial Policy Checklist.

Statistics

For all statistical analyses, confirm that the following items are present in the figure legend, table legend, main text, or Methods section.

n/a	Confirmed	
☐	☒	The exact sample size (*n*) for each experimental group/condition, given as a discrete number and unit of measurement
☐	☒	A statement on whether measurements were taken from distinct samples or whether the same sample was measured repeatedly
☐	☒	The statistical test(s) used AND whether they are one- or two-sided<br>*Only common tests should be described solely by name; describe more complex techniques in the Methods section.*
☒	☐	A description of all covariates tested
☐	☒	A description of any assumptions or corrections, such as tests of normality and adjustment for multiple comparisons
☐	☒	A full description of the statistical parameters including central tendency (e.g. means) or other basic estimates (e.g. regression coefficient) AND variation (e.g. standard deviation) or associated estimates of uncertainty (e.g. confidence intervals)
☐	☒	For null hypothesis testing, the test statistic (e.g. *F*, *t*, *r*) with confidence intervals, effect sizes, degrees of freedom and *P* value noted<br>*Give P values as exact values whenever suitable.*
☒	☐	For Bayesian analysis, information on the choice of priors and Markov chain Monte Carlo settings
☒	☐	For hierarchical and complex designs, identification of the appropriate level for tests and full reporting of outcomes
☐	☒	Estimates of effect sizes (e.g. Cohen's *d*, Pearson's *r*), indicating how they were calculated

Our web collection on statistics for biologists contains articles on many of the points above.

Software and code

Policy information about availability of computer code

Data collection	ChIP-seq and RNA-seq data in GEO were downloaded using Sratoolkit (version 2.10.9) and newly generated ChIP-seq and RNA-seq reads were collected using HCS 3.4.0 software for HiSeq 3000.
Data analysis	FastQC tool (version 0.11.9); Trimmomatic (version 0.36);Bowtie (version 1.2.2); Samtools (version 1.8); Picard (version 2.9.2); Homer (version 4.8.2); DeepTools (version 3.1.3); IGV(version 2.5.3); MACS (version 2.2.7.1); Bedtools (version 2.29.2); STAR RNA-seq (version 2.5.4a); HTSeq (version 0.9.1) ; R (version 3.6.3); Bioconductor (version 3.10); DESeq2; RUVSeq package; dplyr; ggplot2, GraphPad Prism 8 (version 8.2.0)

For manuscripts utilizing custom algorithms or software that are central to the research but not yet described in published literature, software must be made available to editors and reviewers. We strongly encourage code deposition in a community repository (e.g. GitHub). See the Nature Research guidelines for submitting code & software for further information.

Data

Policy information about availability of data

All manuscripts must include a data availability statement. This statement should provide the following information, where applicable:

- Accession codes, unique identifiers, or web links for publicly available datasets
- A list of figures that have associated raw data
- A description of any restrictions on data availability

All data were obtained or uploaded to Gene Expression Omnibus (GEO). ChIP-seq for STATs and CTCF was obtained under GSE31477 and GSE101051, respectively. ChIP-seq data for H3K27ac and H3K4me1 from human lung tissues were downloaded from GSE143115 and 142958. DNase I hypersensitive (DHS) data from human lung tissues and SAECs were obtained under GSE90364 and GSE29692, respectively. RNA-seq data from other types of lung cells, BEAS-2B cells and AT2s, was obtained GSE148829 and GSE152586, respectively. The RNA-seq data and ChIP-seq for SAECs in the presence of cytokines and the JAK inhibitors were uploaded to GSE161665 (ChIP-seq in GSE161663 and RNA-seq in GSE161664).

Field-specific reporting

Please select the one below that is the best fit for your research. If you are not sure, read the appropriate sections before making your selection.

- [x] Life sciences
- [ ] Behavioural & social sciences
- [ ] Ecological, evolutionary & environmental sciences

For a reference copy of the document with all sections, see nature.com/documents/nr-reporting-summary-flat.pdf

Life sciences study design

All studies must disclose on these points even when the disclosure is negative.

Sample size	Sample size was determined based on cell line available for our study. In this manuscript, we used fHuman small airway epithelial cells (SAEC).
Data exclusions	None of data were excluded in the data analysis.
Replication	The number of independent replicates for each experiment is indicated at the corresponding figure legend in the manuscript. In general, at least three independent replicates and two independent ChIP-seq replicates were performed.
Randomization	Cells were cultured at multiple plates with similar concentration and randomly selected for various treatment.
Blinding	Blinding was not applicable to the experiments.

Reporting for specific materials, systems and methods

We require information from authors about some types of materials, experimental systems and methods used in many studies. Here, indicate whether each material, system or method listed is relevant to your study. If you are not sure if a list item applies to your research, read the appropriate section before selecting a response.

Materials & experimental systems

n/a	Involved in the study	
[ ]	[x]	Antibodies
[ ]	[x]	Eukaryotic cell lines
[x]	[ ]	Palaeontology and archaeology
[x]	[ ]	Animals and other organisms
[x]	[ ]	Human research participants
[x]	[ ]	Clinical data
[x]	[ ]	Dual use research of concern

Methods

n/a	Involved in the study	
[ ]	[x]	ChIP-seq
[x]	[ ]	Flow cytometry
[x]	[ ]	MRI-based neuroimaging

Antibodies

Antibodies used	5-10 ug of antibodies were added in 1 mg of total proteins (1ml solution) for ChIP-seq.<br>1:1000 of antibodies were added for western blot.<br>H3K27ac (Abcam, ab4729), RNA polymerase II (Abcam, ab5408), H3K4me1 (Active Motif, 39297) and H3K4me3 (Millipore, 07-473)<br>ACE2 (Proteintech, 21115-1-AP) , GAPDH (Cell signaling, #5174)
Validation	H3K27ac - PMID: 27376239, 30285185, 27694626, 32636391, 28009300, 28714474, 28334928, 32321991, 26446995, 127139, 161620, 161620<br>RNA polymerase II - PMID: 27376239, 30285185, 32636391, 28334928, 32321991, 26446995, 127139, 161620<br>H3K4me1 - PMID: 27215382, 127139, 161620<br>H3K4me3 - PMID: 27376239, 32636391, 28009300, 28714474, 32321991, 26446995, 27215382, 127139, 161620

Eukaryotic cell lines

Policy information about cell lines

Cell line source(s)	Human small airway epithelial cells (SAEC) obtained from Lifeline Technology (FC-0016)
Authentication	The cell line was not authenticated.
Mycoplasma contamination	The line tested was negative for mycoplasma contamination.
Commonly misidentified lines (See ICLAC register)	No commonly misidentified cell lines were used in the study.

ChIP-seq

Data deposition

- [x] Confirm that both raw and final processed data have been deposited in a public database such as GEO.
- [x] Confirm that you have deposited or provided access to graph files (e.g. BED files) for the called peaks.

Data access links<br>*May remain private before publication.*	https://www.ncbi.nlm.nih.gov/geo/query/acc.cgi?acc=GSE161665<br>Secure token: abopugiyhdazncx
Files in database submission	SAEC_Control_H3K4me1_rep1.fastq.gz<br>SAEC_Control_H3K4me1_rep2.fastq.gz<br>SAEC_Control_H3K4me3_rep1.fastq.gz<br>SAEC_Control_H3K4me3_rep2.fastq.gz<br>SAEC_Control_H3K27ac_rep1.fastq.gz<br>SAEC_Control_H3K27ac_rep2.fastq.gz<br>SAEC_Control_PolII_rep1.fastq.gz<br>SAEC_Control_PolII_rep2.fastq.gz<br>SAEC_IFN-b_H3K4me1_rep1.fastq.gz<br>SAEC_IFN-b_H3K4me3_rep1.fastq.gz<br>SAEC_IFN-b_H3K27ac_rep1.fastq.gz<br>SAEC_IFN-b_H3K27ac_rep2.fastq.gz<br>SAEC_IFN-b_PolII_rep1.fastq.gz<br>SAEC_IFN-b_PolII_rep2.fastq.gz<br>SAEC_IFNb_Ruxolitinib_H3K27ac.fastq.gz<br>SAEC_IFNb_Ruxolitinib_PolII.fastq.gz<br>SAEC_Control_H3K4me1_rep1.ucsc.bedGraph.gz.tdf<br>SAEC_Control_H3K4me1_rep2.ucsc.bedGraph.gz.tdf<br>SAEC_Control_H3K4me3_rep1.ucsc.bedGraph.gz.tdf<br>SAEC_Control_H3K4me3_rep2.ucsc.bedGraph.gz.tdf<br>SAEC_Control_H3K27ac_rep1.ucsc.bedGraph.gz.tdf<br>SAEC_Control_H3K27ac_rep2.ucsc.bedGraph.gz.tdf<br>SAEC_Control_PolII_rep1.ucsc.bedGraph.gz.tdf<br>SAEC_Control_PolII_rep2.ucsc.bedGraph.gz.tdf<br>SAEC_IFN-b_H3K4me1_rep1.ucsc.bedGraph.gz.tdf<br>SAEC_IFN-b_H3K4me3_rep1.ucsc.bedGraph.gz.tdf<br>SAEC_IFN-b_H3K27ac_rep1.ucsc.bedGraph.gz.tdf<br>SAEC_IFN-b_H3K27ac_rep2.ucsc.bedGraph.gz.tdf<br>SAEC_IFN-b_PolII_rep1.ucsc.bedGraph.gz.tdf<br>SAEC_IFN-b_PolII_rep2.ucsc.bedGraph.gz.tdf<br>SAEC_IFNb_Ruxolitinib_H3K27ac.ucsc.bedGraph.gz.tdf<br>SAEC_IFNb_Ruxolitinib_PolII.ucsc.bedGraph.gz.tdf
Genome browser session<br>(e.g. UCSC)	UCSC Genome browser

Methodology

Replicates	We generated two biological replicates for each experiments.
Sequencing depth	All Sequencing were done as 51bp single end sequence. Sequencing were done to achieve > 30 million reads per biological replicates .
Antibodies	H3K27ac (Abcam, ab4729), RNA polymerase II (Abcam, ab5408), H3K4me1 (Active Motif, 39297) and H3K4me3 (Millipore, 07-473)
Peak calling parameters	MACS2 was used with default settings for peak calling.
Data quality	We evaluated the data quality by FASTQC, track view and calculation the correlation of different groups.
Software	FastQC tool (version 0.11.9); Trimmomatic (version 0.36);Bowtie (version 1.2.2); Samtools (version 1.8); Picard; Homer (version 4.8.2); DeepTools (version 3.1.3); IGV(version 2.5.3); MACS (version 2.2.7.1); Bedtools (version 2.29.2)
